# The Cure of Carbuncle with Caustic and Strips

**Published:** 1886-04-20

**Authors:** J. McF. Gaston

**Affiliations:** Professor of the Principles and Practice of Surgery in the Southern Medical College, Atlanta, Georgia


### THE CURE OF CARBUNCLE WITH CAUSTIC AND STRIPS.

By J. McF. Gaston, M. D.,
**Professor of the Principles and Practice of Surgery in the Southern Medical College, Atlanta, Georgia.**

The practical importance of recognizing the distinctive characteristics of carbuncle, and adopting appropriate measures of treatment at an early stage of its development, has been strongly impressed upon the medical profession by several notable fatal results of this disease within the past year. Being thoroughly convinced that none subjected to the course pursued by me in the management of this affection, during an extended professional experience, are likely to succumb from this disorder, I am prompted to call the attention of practitioners to the facts observed, not only by myself but by intellegent colleagues, in connection with the simple but effective measures which have secured invariable salutary results.

That my article on "The distinguishing features of malignant pustule and anthrax," published in the September number of Gaillard's Journal for 1881, setting forth pointedly my successful treatment of carbuncle, should have received the endorsement of that accurate and judicious observer, Dr. Simon Baruch, in a subsequent issue of the Journal, warrants me in referring all who are interested to what was styled by him "the able essay of Dr. Gaston." My readers will not, I trust, attribute this allusion to egotism on my part, as a consideration far above any thought of self induces me, at present, to urge upon my colleagues the great benefits to suffering humanity, which must accrue from putting into practice the measures which are therein inculcated, as being uniformly successful in arresting the progress of a most hideous disease.

For the practical exemplification of my mode of proceeding, I am permitted to present the details of a case in the person of the Rev. J. M. White, of this city, who was attended in consultation with Drs. W. R. D. Thompson and G. G. Roy; and members of the profession here and elsewhere may verify the accuracy of my statements by application to either of these gentlemen, personally or by correspondence. At the same time, I may state that Dr. K. C. Divine has carried out my suggestion in the treatment of several cases of carbuncle with the most satisfactory results, being thus in a position to substantiate the efficacy of this mode of treatment. Numerous other cases under my immediate observation might be cited; but preferring to have the corroborative testimony of these disinterested parties, the claims of my treatment to consideration and adoption will be fully appreciated by the result in this case of a gentleman who is so well and favorably known, not only in this city but in other sections of this State and elsewhere in the South.

After a tour of travel in different States, the Rev. John M. White returned to Atlanta with marked aggravation of a local inflammation that had given him trouble for some days, situated over the left lumbar region, at a point about equi-distant from the vertebral column and the lower margin of the ribs. There was a well defined protuberance, with circumscribed induration of the subcutaneous tissues, and at the apex, having several small openings, from which there was some sanious discharge, accompanied by a deepseated pain and a general feeling of discomfort, with sense of lassitude. The constitutional disturbances indicated rather a low order of febrile excitement, with a brownish coating of the tongue, and lack of appetite. All the symptoms, considered in connection with the tumefactions, whose area had a diameter of six inches, and whose surface was of a dusky-red hue, induced us to unite in the diagnosis of anthrax, known ordinarily by the popular designation of carbuncle. I concurred in the suggestions of my colleagues as to the general treatment, and they acquiesced in the measures proposed by me for the topical management of the case, while our joint attendance was continued from day to day. The chief reliance, internally, was upon small doses of the sulphide of calcium during the first week of the treatment, and subsequently upon general tonics. In the meantime the entire surface of the indurated area was well moistened and a stick of lunar caustic passed over it, and, after drying, strips of India-rubber adhesive plaster, long enough to reach across the indurated tissues upon the skin on either side, were drawn tightly over all the carbuncle, so as to produce compression at every point involved in the disease. The cribriform apex was closed in by these strips in common with the other degenerated structures, and over them was laid a compress secured by a bandage around the body. The coating of nitrate of silver over the surface, and the compression by adhesive plaster upon the indurated protuberance, with the application of the compress and bandage, were repeated daily. But after the second day there appeared some signs of suppuration at the apex, and by a crucial arrangement of the strips a small opening was left for it to escape, and this point was covered with some loose charpie for its absorption as it made its escape in the intervals of dressing. After the fourth day the stick of caustic was not passed over the outer surface any more, but was carried within the aperture and around in the cavity so as to detach the disintegrated fibrous substance and connective tissue. On the fifth day suppuration was fairly established, and the discharge was free upon the pressure made in the reapplication of the plaster on all sides of the central opening. The surrounding induration gradually disappeared, and the cavity diminished, under the daily use of compression in this manner, until, at the expiration of one week, there remained no distinctive characteristics of the carbuncle. The crucial arrangement of the strips around the orifice, and extending to the base of whatever induration remained, was kept up for another week, with charpie and basilicon ointment upon the opening, until all discharge ceased. Granulations springing up then from the bottom of the cavity, and the walls being approximated constantly by the compression, there was but little evidence of the destructive process at the end of the third week, from which time our patient entered upon convalescence ; and at the end of the fourth week he was out upon the streets enjoying the pleasing consciousness of complete restoration to health, having resumed his pulpit duties.

Being satisfied, from my observation, that this mode of treating most of the simple cases of carbuncle will secure salutary results in the course of a single week, without any notable cicatrix afterwards, I have to urge its efficacy in even the gravest forms of this serious disorder, as having precedence to any and all other measures of treatment.

There may co-exist with carbuncle various constitutional derangements which require special medication for their correction, and this being accomplished nothing more is needed than the application of nitrate of silver and adhesive plasters.

